# Instantaneous Best Integer Equivariant Position Estimation Using Google Pixel 4 Smartphones for Single- and Dual-Frequency, Multi-GNSS Short-Baseline RTK

**DOI:** 10.3390/s22103772

**Published:** 2022-05-16

**Authors:** Chien Zheng Yong, Ken Harima, Eldar Rubinov, Simon McClusky, Robert Odolinski

**Affiliations:** 1 National School of Surveying, University of Otago, 310 Castle Street, Dunedin 9016, New Zealand; chienzheng.yong@utm.my; 2 Geomatic Innovation Research Group, Faculty of Built Environment and Surveying, Universiti Teknologi Malaysia, Johor Bahru 81310, Johor, Malaysia; 3 Geoscience Australia, GPO Box 378, Canberra, ACT 2601, Australia; ken.harima@ga.gov.au (K.H.); simon.mcclusky@ga.gov.au (S.M.); 4 FrontierSI, Goods Shed, Village Street, Docklands, VIC 3008, Australia; erubinov@frontiersi.com.au; 5 Research School of Earth Sciences, Australian National University, Canberra, ACT 2601, Australia

**Keywords:** best integer equivariant (BIE), smartphone positioning, mean squared error (MSE), real-time kinematic (RTK), multi-GNSS

## Abstract

High-precision global navigation satellite system (GNSS) positioning and navigation can be achieved with carrier-phase ambiguity resolution when the integer least squares (ILS) success rate (SR) is high. The users typically prefer the float solution under the scenario of having a low SR, and the ILS solution when the SR is high. The best integer equivariant (BIE) estimator is an alternative solution since it minimizes the mean squared errors (MSEs); hence, it will always be superior to both its float and ILS counterparts. There has been a recent development of GNSSs consisting of the Global Positioning System (GPS), Galileo, Quasi-Zenith Satellite System (QZSS), and the BeiDou Navigation Satellite System (BDS), which has made precise positioning with Android smartphones possible. Since smartphone tracking of GNSS signals is generally of poorer quality than with geodetic grade receivers and antennas, the ILS SR is typically less than one, resulting in the BIE estimator being the preferred carrier phase ambiguity resolution option. Therefore, in this contribution, we compare, for the first time, the BIE estimator to the ILS and float contenders while using GNSS data collected by Google Pixel 4 (GP4) smartphones for short-baseline real-time kinematic (RTK) positioning. It is demonstrated that the BIE estimator will always give a better RTK positioning performance than that of the ILS and float solutions while using both single- and dual-frequency smartphone GNSS observations. Lastly, with the same smartphone data, we show that BIE will always be superior to the float and ILS solutions in terms of the MSEs, regardless of whether the SR is at high, medium, or low levels.

## 1. Introduction

The key to high-precision global navigation satellite system (GNSS) positioning and navigation is carrier-phase integer ambiguity resolution. The ambiguity-fixed GNSS baseline, as obtained by integer least squares (ILS), is commonly expected to be superior to its float counterpart if the integer ambiguity success rate, i.e., the probability of correct integer estimation, is close to the maximum value of one. On the other hand, the float solution usually becomes the positioning preference when the success rate is too low. Alternatively, the best integer equivariant (BIE) estimator, as introduced by Teunissen [1], can be used since it always provides the optimal solution in terms of the mean squared errors (MSEs). Several studies have already investigated the extremely poor multipath suppression and linearly polarized patch antenna in the smartphone that is the foremost hurdle to achieving centimeter-level accurate positioning [2,3,4,5,6,7,8,9]. Since smartphones track GNSS signals with a poorer quality than geodetic-grade receivers and antennas, the ILS SR is typically different from one, and the BIE estimator would then be the preferred option. Laurichesse et al. [10] used undifferenced ambiguity resolution in precise point positioning (PPP) mode with smartphone measurements. Darugna et al. [9] and Warnant et al. [11] compared the positioning performance of different multi-GNSS positioning techniques (i.e., single-point positioning (SPP), differential GPS (DGPS), and real-time kinematic (RTK)) with different smartphone models. A much more recent study from Paziewski et al. [12] assessed the performance of several Android smartphones in relative positioning, whereby centimeter-level precision was achievable with fixed ambiguities. In this contribution, we study, for the first time, the single-baseline RTK positioning performance of the BIE estimator using smartphone GNSS data. 

Verhagen and Teunissen [13] proved that this estimator is always optimal in terms of the MSE, while Wen et al. [14] demonstrated the use of the BIE estimator for GNSS precise point positioning (PPP). In Brack et al. [15] and Brack [16], a sequential BIE approach was developed. Subsequently, Teunissen [17] extended the theory of integer equivariant estimation by developing the principle of BIE estimation for the class of elliptically contoured distributions, while Odolinski and Teunissen [18] analyzed the BIE performance for low-cost, single- and dual-frequency, short- to long-baseline multi-GNSS RTK positioning, and they found that the BIE positions reveal a ‘star-like’ pattern when the ILS SRs are high. Odolinski and Teunissen [19] recently compared the RTK positioning performance of BIE for the multivariate normal and multivariate t-distribution. 

The recent development of smartphone GNSS chipsets, such as Broadcom BCM47755 embedded, makes precise code-based positioning [20], PPP [21], and instantaneous, centimeter-level RTK positioning possible with Android-based smartphones [2,3,12,22,23,24]. In this contribution, we assess the BIE positioning performance using Google Pixel 4 (GP4) smartphones and compare the performance to that of the ILS and float estimators. The performance of the BIE estimator is assessed from the MSE perspective while using multi-GNSS smartphone data for an instantaneous (single-epoch) and single-baseline RTK model, while employing single- and dual-frequency observations. 

This contribution is organized as follows: in Section 2, we describe the functional model that is employed for instantaneous single-baseline RTK positioning. In this section, we emphasize the differences among the float, ILS, and BIE estimators. In Section 3, we present the smartphone GNSS data and stochastic model settings. The setup configuration deployed in this study is deemed to be the best configuration by having the smartphones placed in an upright position, as examined and proven in Yong et al. [22]. Then, in Section 4, we provide an analysis of the single- and dual-frequency RTK positioning performance under different model strengths. We further analyze the performance of BIE and compare it to the ILS and float contenders from the MSE perspective. Lastly, a summary with conclusions is given in Section 5.

## 2. Instantaneous, Single- and Dual-Frequency, Multi-GNSS RTK Using the Float, Integer Least Squares, and Best Integer Equivariant Estimators

In this section, we describe the functional model employed for the single-baseline RTK model while tracking single- and dual-frequency multi-GNSS observations using GP4 smartphones. We also introduce the float, ILS, and BIE estimators.

### 2.1. Functional Model

We assume that the two GP4 smartphones track GPS, Galileo, QZSS, and BDS code and carrier-phase frequencies on two frequencies. We make use of broadcast ephemerides for satellite orbits and clocks. The relative ionospheric, tropospheric delays and satellite orbit errors can be assumed negligible, since we employ short baselines. The single-epoch (instantaneous) and linearized double-differenced (DD) system of observation equations reads as follows:

(1) y=Aa+Bb,

where y is the vector of DD carrier-phase and code observations, A is the design matrix of the DD integer ambiguities in vector a, and B corresponds to the design matrix of the real-valued baseline components b. We employ system-specific reference satellites when performing the between-satellite single-differences. We note that, if we would take a common reference satellite on the overlapping frequencies between the systems, it could further strengthen the model [25,26]. For the stochastic model, we use an elevation weighting sine function as employed in RTKlib v2.4.3 [27].

### 2.2. Float Estimation

To obtain the float solution, denoted with a ‘hat’ symbol, we estimate the ambiguities and baseline components as real valued parameters in a standard least-squares solution, obtaining

(2) $$[\hat{a,}\hat{b}{]}^{T},\;\left[\begin{array}{cc} Q_{\hat{a}\hat{a}} & Q_{\hat{a}\hat{b}}\\ Q_{\hat{b}\hat{a}} & Q_{\hat{b}\hat{b}} \end{array}\right],$$

where $\hat{a},\hat{b}$ are the vectors of the float ambiguities and baseline components with dimensions *n* and p, respectively, $Q_{\hat{a}\hat{a}}$, $Q_{\hat{b}\hat{b}}$ denote the corresponding variance covariance (VCV) matrices, and $Q_{\hat{a}\hat{b}}=Q_{\hat{b}\hat{a}}^{T}$ are the corresponding covariance matrices between the ambiguities and baseline components.

### 2.3. Integer Least-Squares Estimation

By using the float ambiguities $\hat{a}$ in Equation (2) we can find the integer least-squares solution of the ambiguities, denoted with a ‘check’ symbol, by solving the following problem:

(3) $$\overset{ˇ}{a}=\arg\min||\hat{a}-z|{|}_{Q_{\hat{a}\hat{a}}}^{2},$$

where $|\left|.\right|{|}_{Q_{\hat{a}\hat{a}}}^{2}={\left(.\right)}^{T}Q_{\hat{a}\hat{a}}^{-1}\left(.\right)$. This ILS problem is efficiently solved using the LAMBDA (least-squares ambiguity decorrelation adjustment) method [28], finally yielding the following fixed baseline solution:

(4) $$\overset{ˇ}{b}=\hat{b}-Q_{\hat{b}\hat{a}}Q_{\hat{a}\hat{a}}^{-1}\left(\hat{a}-\overset{ˇ}{a}\right).$$

The corresponding VCV matrix, provided that the uncertainty in $\overset{ˇ}{a}$ can be neglected, reads

(5) $$Q_{\overset{ˇ}{b}\overset{ˇ}{b}}=Q_{\hat{b}\hat{b}}-Q_{\hat{b}\hat{a}}Q_{\hat{a}\hat{a}}^{-1}Q_{\hat{a}\hat{b}}.$$

The precision of the fixed ILS baseline $\overset{ˇ}{b}$ in Equation (4) is driven by the very precise phase data provided that the ILS SR is sufficiently high, while in the single-epoch case the float solution $\hat{b}$ in Equation (2) is driven by the precision of the code data. This means, in the case that the ILS SR is sufficiently high, that the ILS solution is expected to have at least a two-order of magnitude positioning precision improvement compared to that of the float solution.

### 2.4. Best Integer Equivariant Estimation

When the ILS SR is low, the user will normally prefer the float solution $\hat{b}$ in Equation (2) rather than the ILS solution $\overset{ˇ}{b}$ in Equation (4). The alternative is to use the BIE estimator to solve for the ambiguities [1]. Assuming normally distributed GNSS data, the BIE estimator we use is denoted with an ‘overline’ symbol and reads

(6) $${\bar{a}}^{\lambda }=\underset{z\epsilon \Theta_{\hat{a}}^{\lambda }}{\sum }z\frac{\exp\left(-\frac{1}{2}||\hat{a}-{z||}_{Q_{\hat{a}\hat{a}}}^{2}\right)}{\sum_{z\epsilon \Theta_{\hat{a}}^{\lambda }}\exp\left(-\frac{1}{2}||\hat{a}-{z||}_{Q_{\hat{a}\hat{a}}}^{2}\right)},$$

Note in Equation (6) that the BIE solution is not always necessarily an integer as it is a weighted sum of integers. We also remark that, for the BIE estimator, no ratio test is needed [1].

The BIE baseline solution can then be derived as

(7) $$\overset{ˇ}{b}=\hat{b}-Q_{\hat{b}\hat{a}}Q_{\hat{a}\hat{a}}^{-1}\left(\hat{a}-{\bar{a}}^{\lambda }\right),$$

where $\overset{ˇ}{a}$ in Equation (4) is replaced by ${\bar{a}}^{\lambda }$ in Equation (6). Note that the true BIE estimator involves an infinite weighted sum over the whole space of integers for z, which is computationally impractical. Hence, in Equation (6) we make use of a finite integer set $\Theta_{\hat{a}}^{\lambda }$ instead [29], which can be defined as follows:

(8) $$||\hat{a}-z{||}_{Q_{\hat{a}\hat{a}}}^{2}<\lambda^{2},$$

where the threshold $\lambda^{2}$ can be determined from a central chi-squared distribution $\chi^{2}$ with n degrees of freedom and a small significance level $\alpha =10^{-9}$. Note that, for very weak models, the number of candidates within this threshold in Equation (8) can reach several tens of thousands of candidates, whereas, for stronger models, at most a few candidates are usually obtained.

## 3. Google Pixel 4 Smartphone GNSS Data Collection

This section describes the short-baseline setup configurations of the GP4 smartphones while using (1) external antennas and (2) internal smartphone antennas. In this section, we also outline the stochastic model settings.

### 3.1. Setup Configuration with External and Internal Smartphone Antennas 

**Figure 1:**
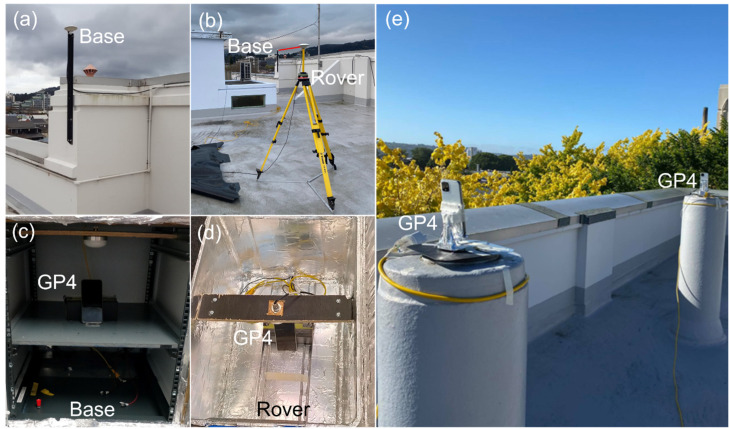
GP4 smartphones in a short-baseline setup configuration with external antennas (**a**,**b**) inside an RF shielding box (**c**,**d**), and with the smartphone internal antennas (**e**) on the rooftop of the building of the School of Surveying in Dunedin, New Zealand.

Figure 1 depicts the external and internal antenna setups to evaluate the positioning performance of the GNSS data observed by GP4 smartphones. The GP4 smartphones are capable of tracking dual-frequency GPS L1 + L5, Galileo E1 + E5a, QZSS L1 + L5, and BDS B1 code and carrier-phase observations. The GP4 smartphones logged the GNSS measurements at a 1-s measurement interval via the Geo++ RINEX Logger vers. 2.1.6.

When evaluating the performance using external antennas, the GP4 smartphones are placed in two separate radiofrequency (RF) shielding boxes to avoid them from receiving the GNSS signals other than from the dedicated reradiating antenna (see Figure 1a–d). The GNSS signals are collected from two distinct active low-cost antennas, Swift GPS500, and then reradiated via a passive antenna inside the RF shielding boxes. The signal amplifier is connected between the rooftop antenna and reradiating antenna to mitigate the effect of signal attenuation over a 30 m LMR-400 flexible low loss communication coaxial cable. A similar setup was validated in Yong et al. [22] that benchmarked the smartphones with survey-grade receivers, to assure that no GNSS signal leakage was experienced in the RF shielding box. The duty-cycling settings of the GP4 smartphones were disabled during the experiment to obtain continuous carrier-phase observations [3].

In addition to the short baseline with external antennas, we assessed the positioning performance of the short baseline while using the smartphone internal antennas (see Figure 1e). Note that the built-in antennas of the GP4 smartphones have been found to be sensitive to poor quality GNSS signals and the surrounding environment [7,22], which means that we can expect larger multipath errors to be present for this setup.

### 3.2. Stochastic Model Settings

The stochastic model was determined by fitting the empirical 95% confidence interval/ellipses to the formal counterparts, as derived from the corresponding VCV matrices of the positions. The empirical VCV matrix was estimated from the positioning errors obtained by comparing the estimated positions to very precise benchmark coordinates, whereas the formal VCV matrix was obtained by the average of all single-epoch formal VCV matrices of the entire observation time span [30]. We used independent datasets to analyze and to formulate the stochastic model for the subsequent sections, and the stochastic model settings were determined for different elevation cut-off angles to formulate the most realistic stochastic models possible. By using realistic stochastic models, we could assure that we obtained the best possible ambiguity resolution and positioning performance results. The different elevation cut-off angles were used to mimic situations in urban canyon environments or when low-elevation multipath is present.

Table 1 depicts the range of the undifferenced and zenith-referenced standard deviations (STDs) utilized in the stochastic models, together with the observation span of the external and internal antenna setup configurations in Figure 1. Note that each GNSS and/or frequency had equal weighting in this article, similar to the RTKlib implementation [27].

Table 1 shows that the code STDs improved by a factor of approximately five when external antennas were used instead of the internal smartphone antennas that were more sensitive to multipath. For example, the code STD increased from a maximum of 1.4 m when using external antennas to approximately 6.0 m when internal antennas were used. The corresponding phase STDs increased from a maximum of 2 mm to 4 mm when using internal antennas. Note that the ambiguity resolution performance in the subsequent sections is driven by the number of satellites and frequencies used, as well as the stochastic model.

## 4. Instantaneous, Short-Baseline, Single- and Dual-Frequency RTK and BIE Positioning with Google Pixel 4 Smartphones

In this section, we investigate the BIE estimator and compare the performance to that of the commonly used ILS and float estimators. The benefit of using the BIE estimator is that the MSEs are always smaller than or at least as good as the float and ILS counterparts. For instance, in practice, when the ILS success rate is lower than the desired 100%, the user usually opts for the float solution when, in fact, BIE would be the preferred option. 

The number of correctly fixed epochs, used below, was determined by the number of epochs where the estimated local east, north, and up coordinate errors were all below or equal to 0.05 m. The ILS success rate was then computed as follows:

(9) $$P_{S_{E}}=\frac{\#\;\mathrm{of}\;\mathrm{correctly}\;\mathrm{fixed}\;\mathrm{epochs}}{\mathrm{total}\;\#\;\mathrm{of}\;\mathrm{epochs}}\times 100\%.$$

In the results below, we investigate the BIE performance for GP4 smartphones when using both external and internal smartphone antennas while collecting single- and dual-frequency multi-GNSS data. 

### 4.1. BIE with External Antennas for Single-Frequency RTK

**Figure 2:**
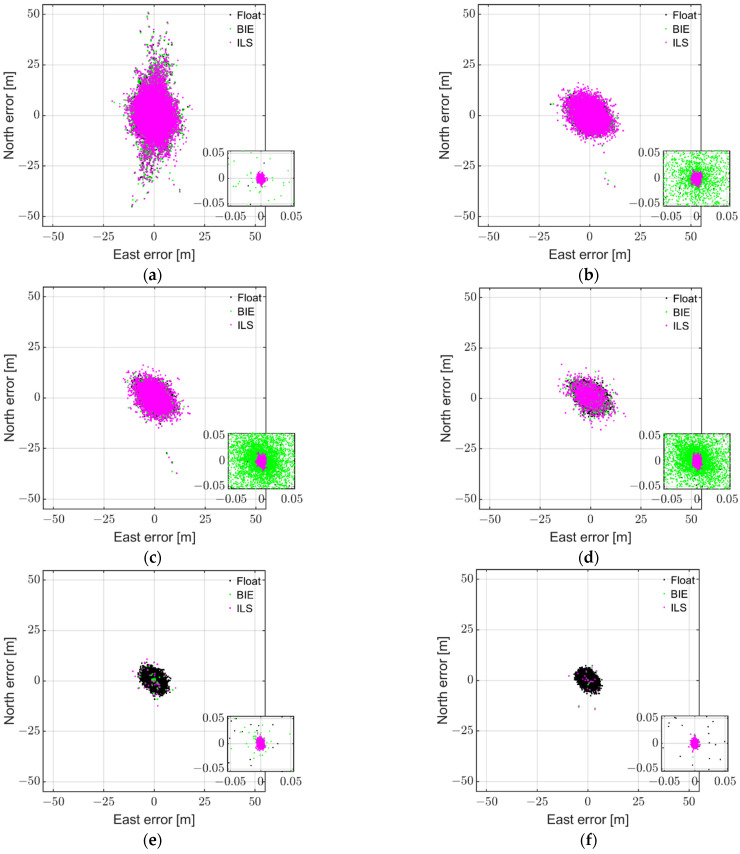
Horizontal (north/east) scatter plot of the multi-GNSS, single-frequency (L1 GPS + E1 Galileo + L1 QZSS + B1 BDS) GP4 data derived with the BIE (*green dots*), ILS (*magenta dots*), and ambiguity float (*black dots*) estimators for instantaneous RTK positioning with external antenna for a short baseline in Dunedin, New Zealand, based on 8 h of data (1 s measurement interval): (**a**) 11.5% ILS SR (38° cut-off); (**b**) 54.9% ILS SR (35° cut-off); (**c**) 79.7% ILS SR (32° cut-off); (**d**) 94.3% ILS SR (30° cut-off); (**e**) 99.8% ILS SR (20° cut-off); (**f**) 99.9% ILS SR (10° cut-off).

Figure 2 depicts the float (black), ILS (magenta), and BIE (green dots) horizontal RTK positioning errors using GP4 smartphones in a short-baseline RTK setup, while using low-cost external antennas. The positioning errors were determined by comparing the estimated positions to very precise benchmark coordinates. These benchmark coordinates were determined using geodetic GNSS receivers and antennas, a Kalman filter, and a multi-epoch model while assuming the ambiguities to be time-constant. Any phase center offsets and variations of the smartphones were neglected in this process. From top to bottom rows and left to right columns, we depict the results for various elevation cut-off angles resulting in ILS SRs of 11.5%, 54.9%, 79.7%, 94.3%, 99.8%, and 99.9%, respectively. We depict L1+ E1 + L1 + B1 GPS + Galileo + QZSS + BDS results, where the zoom-in windows show at least a two-order-of-magnitude improvement when going from ambiguity float and incorrectly fixed ILS solutions to that of the correctly fixed ILS positioning errors. Note that the float solutions, as depicted by black dots, become more precise as the model strength increases.

Figure 2 (top row and left column) shows that many of the incorrectly fixed ILS solutions (magenta dots) are at the meter level, and that the BIE solutions (green dots) resemble the float solutions (black dots underneath the green dots). When the ILS SRs increased, however, such as in the right column and second row, we could see BIE solutions starting to outperform their float counterparts, with a much larger density of BIE solutions with millimeter- to centimeter-level positioning precisions, as shown in the zoom-in windows. In the second row and right column, as well as in the third row, we can further see ILS solutions with larger positioning errors than BIE even though the ILS SRs ranged from 94.3% to 99.9%. 

**Figure 3:**
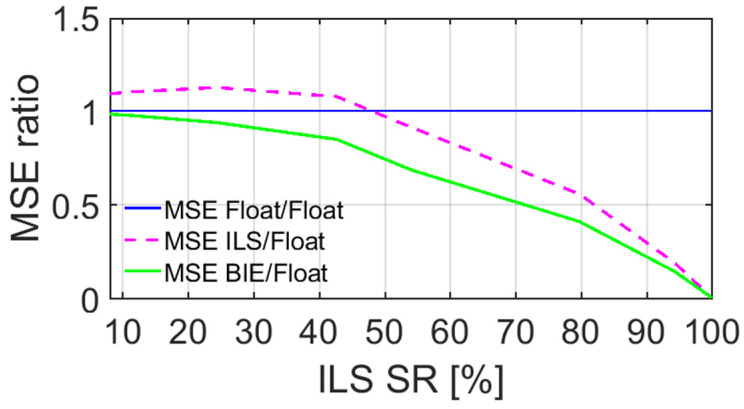
MSE ratios of GP4 single-frequency, short-baseline, and instantaneous RTK positioning errors using external antennas, with BIE (green line), ILS (dashed magenta line), and ambiguity float (blue line), all versus the float solutions.

To further illustrate the optimal performance of the BIE estimator in terms of the positioning MSEs, we depict in Figure 3 the MSE ratios, with respect to the float MSEs, as a function of the ILS SRs. Note that the MSE is here the sum of the variances of the east, north, and up errors, since our estimated positions are unbiased. The float MSE ratio is equal to one and is depicted as a full blue line, whereas the ILS and BIE counterparts are depicted as dashed magenta and full green lines, respectively. Note that these MSE ratio results resemble those of Odolinski and Teunissen [19], albeit based on completely different datasets (smartphone vs. low-cost RTK receiver data).

Figure 3 shows, as expected, that the BIE MSE ratio is equal to that of the float solutions when the ILS SR is close to 0%, and that BIE is equal to ILS when the ILS SR is close to 100%. Most importantly and for all other cases, we can see that the BIE MSE ratios are smaller than those of the float and ILS solutions, respectively. This shows that using the BIE estimator on smartphone data for RTK positioning will give the optimal positioning performance, as measured by the MSEs. 

**Figure 4:**
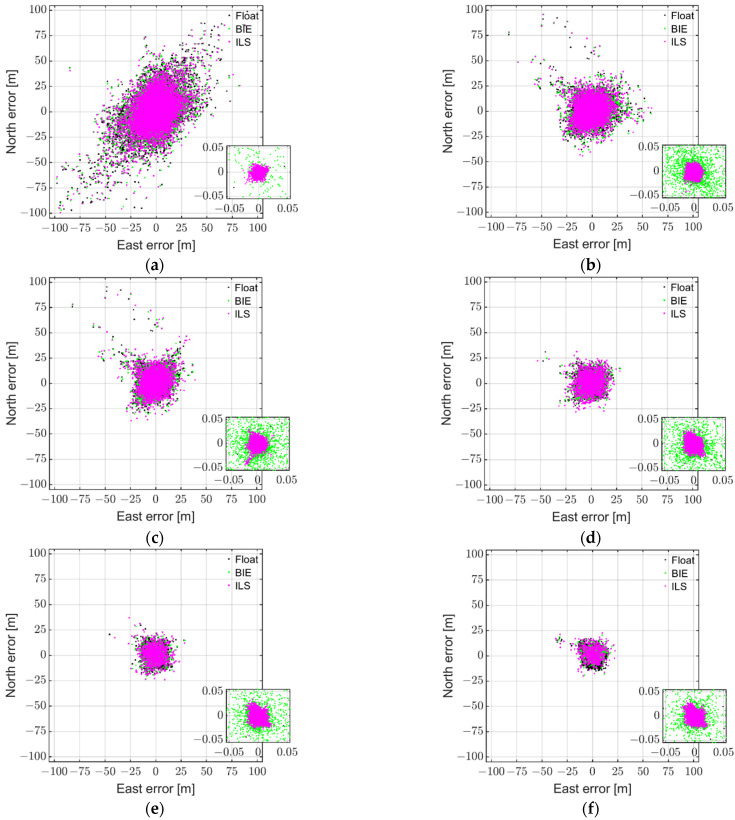
Horizontal (north/east) scatter plot of the multi-GNSS, dual-frequency (L1 + L5 GPS, E1 + E5a Galileo, L1 + L5 QZSS, and B1 BDS) GP4 data derived with the BIE (*green dots*), ILS (*magenta dots*), and ambiguity float (*black dots*) estimators for instantaneous RTK positioning with smartphone internal antennas for a short baseline in Dunedin, New Zealand, based on 6 h of data (1 s measurement interval): (**a**) 9.6% ILS SR (40° cut-off); (**b**) 53.9% ILS SR (30° cut-off); (**c**) 72.1% ILS SR (25° cut-off); (**d**) 84.0% ILS SR (20° cut-off); (**e**) 91.9% ILS SR (15° cut-off); (**f**) 95.4% ILS SR (10° cut-off).

Table 2 shows the percentage of the 3D position errors within a range of 0.05 m, 1 m, 2 m, and 4 m. The given percentages can provide a practical understanding of the distribution of the RTK positioning errors. The largest percentages for each scenario are shown in bold to distinguish the values from each other when rounded to two decimal places. The percentage of the ILS for the weakest model with the lowest ILS SR of 11.52% shows that the BIE solutions are similar to the float solutions. For the strongest model with the highest ILS SR of 99.92%, the results show that BIE resembled the ILS solutions. For all other scenarios, it becomes clear that BIE outperformed both the ILS and the float solutions in terms of not obtaining very large positioning errors, while also having a smaller likelihood than ILS of very small positioning errors (unless the ILS SR is very high). These results are similar to the cumulative distribution functions (CDFs) as discussed in Odolinski and Teunissen ([18]; Figure 4) and Verhagen and Teunissen ([13]; Figure 1).

To also illustrate the corresponding positioning precisions of the different estimators, Table 2 depicts the positioning standard deviations (STDs), the mean number (#) of satellites, and the employed elevation cut-off angles. We can see in Table 2 that the BIE and float solutions have similar STDs for the east, north, and up components when the ILS SR is 11.5% (with a slightly better performance for the BIE estimator), and that both solutions have STDs that are much better than their ILS counterparts. When the ILS SR increased to 54.9%, we can further see that the BIE estimator start to significantly outperform both the float and the ILS solutions, with STDs in east, north, and up of 2.344 m, 2.217 m, and 7.797 m, respectively. The corresponding STDs for the float and ILS solutions are 2.791 m, 2.605 m, and 9.455 m, and 2.661 m, 2.642 m, and 8.990 m in east, north and up, respectively. In other words, the BIE solutions have STDs that are up to more than 1 m smaller (in the up component) than the ILS counterparts.

Lastly, we can see in Table 2 that the BIE solutions have a better performance than their ILS counterparts when the ILS SRs reached values of 99.8% and 99.9%, with smaller positioning STDs by up to several centimeters in east, north, and up, as well as better performance by even several meters than the float solutions. This implies that when the ILS SR is different from the desirable 100%, the BIE estimator will indeed outperform the float and ILS estimators, and this is true even when smartphone GNSS data are used.

### 4.2. BIE with Internal Antennas for Dual-Frequency RTK

In this section, we investigate the corresponding BIE performance when the internal antennas of the smartphones are used. Since, with the smartphone internal antennas, the multipath errors are more significant than when external antennas are used [22], in this section, we use dual-frequency L1 + L5 GPS, E1 + E5a Galileo, L1 + L5 QZSS, and B1 BDS observations to further strengthen the model.

Figure 4 depicts, as in Figure 2, the float (black), ILS (magenta), and BIE (green dots) horizontal RTK positioning errors using GP4 smartphones in a short-baseline RTK setup, but while using the internal antennas of the smartphones. From top to bottom rows and left to right columns, we depict the results for various elevation cut-off angles resulting in ILS SRs of 9.6%, 53.9%, 72.1%, 84.0%, 91.9%, and 95.4%, respectively. The zoom-in windows show at least a two-order-of-magnitude improvement when going from ambiguity float and incorrectly fixed ILS solutions to that of the correctly fixed ILS positioning errors. Note again that, as the model become stronger, the float solutions, as depicted by black dots, become more precise. We also remark here that it is evident that the float and incorrectly fixed ILS solutions have a much poorer precision than in Figure 2. This degradation in precision when internal antennas are used is indeed due to their sensitive to multipath effects, where the code observations, which dominate the precision of the single-epoch float solutions, are more affected [31].

Figure 4 shows, similar to Figure 2, that many of the incorrectly fixed ILS solutions (magenta dots) have errors at the meter level, and that the BIE solutions (green dots) more or less resembles the float solutions (black dots underneath the green dots) when the models are weak (at the top row). When the ILS SRs increases, however, such as in the right column and second row, we can again see that BIE solutions start to outperform their float counterparts, with a much larger density of BIE solutions with millimeter- to centimeter-level positioning precisions as shown in the zoom-in windows. In the second row and right column, as well as in the third row, we can again see ILS solutions with larger positioning errors than BIE despite the fact that the ILS SRs ranged from 84.0% to 95.4%. 

**Figure 5:**
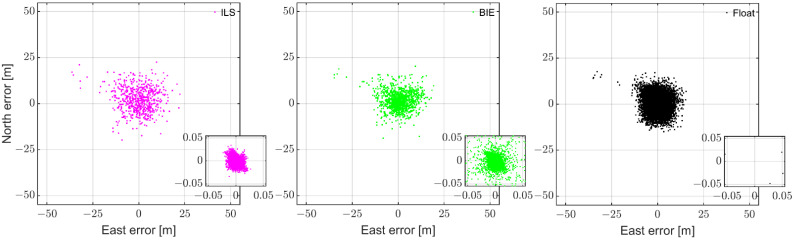
The 95.4% ILS SR scenario (see Figure 4f): horizontal (north/east) scatter plot of the multi-GNSS, dual-frequency (L1 + L5 GPS, E1 + E5a Galileo, L1 + L5 QZSS, and B1 BDS) GP4 data derived with the ILS (left column), BIE (middle column), and ambiguity float (right column) estimators for instantaneous RTK positioning with smartphone internal antennas.

To show the above superior performance of the BIE estimator in a different way, Figure 5 illustrates the 95.4% ILS SR scenario (see Figure 4f), but now with each solution in a separate subplot. The figure shows that the BIE solutions are indeed superior to their float and ILS counterparts, with a better precision than both estimators and fewer large positioning errors than the ILS estimator.

**Figure 6:**
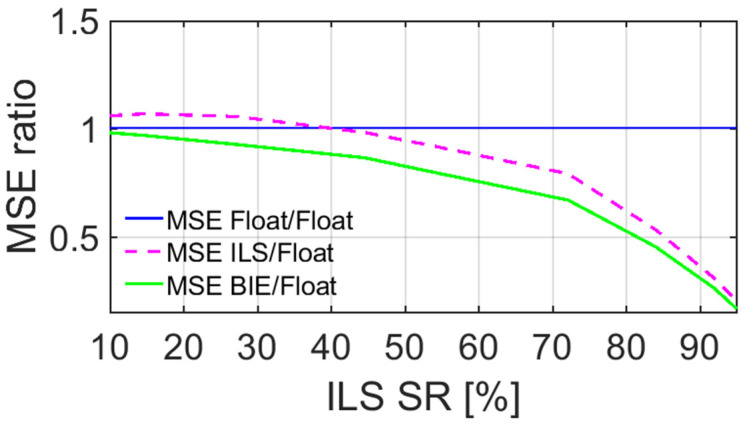
MSE ratios of the GP4 dual-frequency, short-baseline, and instantaneous RTK positioning errors using internal antennas, with BIE (green line), ILS (dashed magenta line), and ambiguity float (blue line), all versus the float solutions.

To again illustrate the optimal BIE performance, we depict in Figure 6 the MSE ratios, with respect to the float MSEs, as a function of the ILS SRs. The float MSE ratio is depicted as a full blue line, whereas the ILS and BIE counterparts are depicted as dashed magenta and full green lines, respectively. Figure 6 shows, similar to Figure 3, that the BIE MSE ratio is equal to that of the float solutions when the ILS SR is close to 0%, and that BIE is close to ILS when the ILS SR is also close to 100% (i.e., 95.4%). Most importantly and for all other cases, we can again see that the BIE MSE ratios are smaller than those of the float and ILS solutions. This shows that using the BIE estimator on smartphone data for RTK positioning, even when the internal smartphone antennas are used, will give the optimal positioning performance. 

Table 3 depicts the corresponding percentage of the 3D position errors within a range of 0.05 m, 1.0 m, 2.0 m, and 4.0 m, the positioning standard deviations (STDs), the mean number (#) of satellites, and the employed elevation cut-off angles when the internal smartphone antennas are used. We can again see that the percentages of the position errors are consistent with the CDFs in, e.g., Figure 4 of Odolinski and Teunissen [18]. Similarly, the BIE solutions have always smaller STDs for the east, north, and up components when the ILS SR is between 9.6% and 95.4%, with better STDs by more than 1 m to several tens of centimeters than the ILS solutions in east, north, and up, and better performance by several meters than the float solutions when the ILS SR is high. This implies again that when the ILS SR is different from the desirable 100%, the BIE estimator will indeed outperform the float and ILS estimators, and this is true even when smartphone GNSS data with internal antennas are used.

## 5. Discussion

In this contribution, we analyzed the best integer equivariant (BIE) estimator for real GNSS data collected by Google Pixel 4 (GP4) smartphones and antennas. We compared the instantaneous (single-epoch) positioning performance of BIE to the float and integer least squares (ILS) estimators that are commonly used when the ILS success rate (SR) is different from one and close to one, respectively. Radiofrequency (RF) shielding boxes and reradiating antennas were used to track GNSS signals from external low-cost antennas, consisting of L1 + L5 GPS, E1 + E5a Galileo, L1 + L5 QZSS, and B1 BDS code and carrier-phase observations. The short-baseline real-time kinematic (RTK) performance was also evaluated while using the GP4 internal smartphone antennas. We investigated the BIE performance both when single-frequency and dual-frequency measurements were employed for the combination of GPS + Galileo + QZSS + BDS. We showed that the BIE positioning performance was superior to that of the ILS and float estimators when the ILS SR is different from one. This was demonstrated to be true on the basis of real multi-GNSS data collected by the GP4 smartphones and antennas.

Our BIE performance evaluation consisted of comparing the estimated positions to very precise benchmark coordinates, and the optimality of the BIE estimator was further evaluated through its position mean squared errors (MSEs) and standard deviations (STDs). It was shown that the BIE performance resembles that of the float estimator when the ILS SR is very low and was similar to that of the ILS when the ILS SR is very high. For all other cases, we demonstrated that BIE outperformed both the float and the ILS estimators even when on the basis of real GP4 smartphone data while using external and internal smartphone antennas. Future studies could involve evaluating the GP4 smartphone BIE RTK positioning performance for longer baselines, when the relative atmospheric delays need to be estimated [19].

## Figures and Tables

**Table 1:** Stochastic model settings in terms of undifferenced and zenith-referenced STD range (minimum to maximum) of the code and phase observations. In the last column, the time span of the data to be analyzed is also depicted (in universal coordinated time, UTC).

Antennas Used	Range of Phase STDs (m)	Range of Code STDs (m)	DoY (Hours of Day), hh:mm:ss UTC
External antenna	0.001–0.002	1.198–1.413	228–2021 (8 h), 13:35:00–21:34:59
Internal antenna	0.003–0.004	5.985–5.998	344 and 345–2020 (6 h), 21:03:00–02:57:59

**Table 2:** Empirical STDs of the ILS, BIE, and float solutions for single-frequency and instantaneous RTK using GP4 smartphones, based on 8 h observations for a short-baseline external antenna data experiment (see Figure 2). Comparisons of the percentage of the 3D position errors within a range of 0.05 m, 1.0 m, 2.0 m, and 4.0 m are also given. Bold values show the largest percentages for each scenario and estimator.

Mode	ILS Success Rate (%)	Percentage of the 3D Position Errors (%)				STD (m)			Mean # of Satellites	Elevation Cut-Off Angle
		≤0.05 m	<1.0 m	<2.0 m	<4.0 m	E	N	U		
ILS	11.52	**11.52**	46.12	69.27	91.18	3.509	5.624	15.044	8.6	
BIE		0.09	**46.90**	**72.71**	**93.47**	3.238	5.283	14.170		38
Float		0.00	44.70	71.34	93.34	3.282	5.332	14.358		
ILS	54.92	**54.92**	**72.79**	85.08	96.16	2.661	2.642	8.990	10.6	
BIE		5.49	70.92	**88.25**	**97.89**	2.344	2.217	7.797		35
Float		0.00	55.65	82.10	97.55	2.791	2.605	9.455		
ILS	79.75	**79.75**	**87.32**	92.86	98.01	1.849	1.883	6.035	11.7	
BIE		29.05	85.97	**94.54**	**98.92**	1.634	1.596	5.172		32
Float		0.00	58.45	84.82	98.17	2.507	2.462	8.096		
ILS	94.29	**94.29**	**96.38**	97.85	99.35	1.126	1.024	3.073	13.5	
BIE		67.81	95.86	**98.27**	**99.56**	1.014	0.887	2.667		30
Float		0.00	60.59	86.41	98.64	2.370	2.258	7.147		
ILS	99.85	**99.85**	**99.90**	99.94	99.99	0.183	0.192	0.425	17.9	
BIE		99.65	99.90	**99.95**	**99.99**	0.170	0.179	0.400		20
Float		0.00	70.98	93.41	99.78	1.846	1.865	4.762		
ILS	99.92	**99.92**	**99.97**	99.98	99.99	0.095	0.147	0.444	23.6	
BIE		99.91	99.97	**99.98**	**99.99**	0.093	0.143	0.440		10
Float		0.00	78.03	96.81	99.98	1.556	1.633	3.411		

**Table 3:** Empirical STDs of the ILS, BIE, and float solutions for dual-frequency and instantaneous RTK using GP4 smartphones, based on 6 h observations for a short-baseline internal antenna experiment (see Figure 4). Comparisons of the percentage of the 3D position errors within a range of 0.05 m, 1.0 m, 2.0 m, and 4.0 m are also given. Bold values show the largest percentages for each scenario and estimator.

Mode	ILS Success Rate (%)	Percentage of the 3D Position Errors (%)				STD (m)			Mean # of Satellites	Elevation Cut-Off Angle
		≤0.05 m	<1.0 m	<2.0 m	<4.0 m	E	N	U		
ILS	9.62	**9.62**	**29.28**	44.44	65.86	10.9938	12.0972	29.7068	7.79	
BIE		0.52	27.66	**45.26**	**67.95**	10.6723	11.7231	28.5620		40
Float		0.00	24.19	41.98	66.46	10.8387	11.8522	28.8412		
ILS	53.89	**53.89**	**64.89**	**72.67**	84.46	6.1821	6.1096	15.9402	11.87	
BIE		24.98	60.47	72.50	**86.15**	5.8795	5.5962	14.8443		30
Float		0.00	31.84	54.61	79.30	6.4436	6.4693	16.6158		
ILS	72.15	**72.15**	**78.87**	**83.63**	90.86	4.6011	4.9372	10.8364	13.74	
BIE		46.22	73.89	82.53	**91.38**	4.3570	4.5332	9.9186		25
Float		0.00	36.32	60.89	84.86	5.2655	5.7106	12.0913		
ILS	84.01	**84.01**	**88.10**	**91.31**	95.32	3.0321	3.0510	7.3630	15.49	
BIE		65.93	86.18	90.95	**95.72**	2.8652	2.7173	6.8088		20
Float		0.00	40.10	65.29	88.75	4.2646	4.5909	9.8794		
ILS	91.90	**91.90**	**94.29**	**96.01**	98.05	1.9998	2.0644	4.1177	17.56	
BIE		80.03	93.52	95.99	**98.29**	1.8819	1.8900	3.7877		15
Float		0.00	45.57	71.17	92.70	3.6434	4.0713	7.1847		
ILS	95.39	**95.39**	**96.64**	97.68	98.76	1.5967	1.3888	2.7278	19.81	
BIE		88.37	96.35	**97.75**	**99.07**	1.4831	1.2386	2.4483		10
Float		0.00	47.81	74.55	94.41	3.3856	3.6980	6.1203		

## Data Availability

The broadcast ephemerides were used for satellite orbits and clocks. The Google Pixel 4 smartphone observation data are stored at the School of Surveying data facilities, the University of Otago, and can be made available upon request by contacting the corresponding author C.Z.Y. via email.
